# Association of thyroid function with insulin resistance: data from two population-based studies

**DOI:** 10.1530/ETJ-21-0063

**Published:** 2022-01-27

**Authors:** Dominik Spira, Nikolaus Buchmann, Marcus Dörr, Marcello R P Markus, Matthias Nauck, Sabine Schipf, Joachim Spranger, Ilja Demuth, Elisabeth Steinhagen-Thiessen, Henry Völzke, Till Ittermann

**Affiliations:** 1 Department of Endocrinology and Metabolism, Charité-Universitätsmedizin Berlin, corporate member of Freie Universität Berlin, Humboldt-Universität zu Berlin, and Berlin Institute of Health, Berlin, Germany; 2 Department of Cardiology, Charité-Universitätsmedizin Berlin, corporate member of Freie Universität Berlin, Humboldt-Universität zu Berlin, and Berlin Institute of Health, Berlin, Germany; 3 Department of Internal Medicine B, University Medicine Greifswald, Greifswald, Germany; 4 German Centre for Cardiovascular Research (DZHK), partner site Greifswald, Greifswald, Germany; 5 Institute of Clinical Chemistry and Laboratory Medicine, University Medicine Greifswald, Greifswald, Germany; 6 Institute for Community Medicine, University Medicine Greifswald, Greifswald, Germany; 7 Charité – Universitätsmedizin Berlin, BCRT – Berlin Institute of Health Center for Regenerative Therapies, Berlin, Germany

**Keywords:** thyroid function, insulin resistance, diabetes, lifespan, age

## Abstract

**Objective:**

Thyroid dysfunction is associated with relevant disturbances in glucose metabolism. Moreover, thyroid function undergoes important changes with ageing. The objective of this study was to investigate the association of thyroid function with insulin resistance with particular consideration of possible age-related effect modifications.

**Design:**

A sample of 4193 participants from two independent epidemiological studies, the Study of Health in Pomerania-TREND-0 and the Berlin Aging Study II, was included in this cross-sectional analysis.

**Methods:**

Insulin resistance was estimated by homeostasis model of insulin resistance (HOMA-IR) and the insulin sensitivity index (ISI). Associations of thyroid biomarkers (thyroid-stimulating hormone, free thyroxine, and free triiodothyronine (fT3)) with parameters of glucose metabolism were analysed by regression models adjusted for age, sex, smoking status, and study site.

**Results:**

A higher fT3 was significantly associated with higher fasting glucose and higher fasting and 2-h postload insulin levels, a higher HOMA-IR, and lower ISI. A higher fT3 was also associated with a higher risk for impaired fasting glucose (RR 1.09, 95 CI 1.02; 1.18; *P*  = 0.017). Many of these associations between thyroid markers and parameters of glucose metabolism were significant in young and middle-aged participants but not in older individuals.

**Conclusions:**

The main finding of this study was a consistent association of fT3 with almost all markers of insulin resistance. However, this effect seems to be wearing off in higher age highlighting a potential age-related modification of the interaction between thyroid function and glucose metabolism. Further studies are needed to clarify causal relationships.

## Introduction

Thyroid hormones regulate important metabolic processes. Thus, thyroid dysfunction can lead to clinically relevant alterations in energy expenditure, body weight regulation, glucose metabolism, and lipid metabolism (1). It is well known that thyroid hormones, namely free thyroxine (fT4) and free triiodothyronine (fT3), can exert both insulin agonistic and antagonistic actions in different organs (2). The most profound alterations in glucose metabolism are found in overt hyper- and hypothyroidism. Mechanisms included in hyperthyroidism are an increased hepatic glucose output through a higher rate of gluconeogenesis and glycogenolysis induced by thyroid hormones. Usually, hyperthyroidism also leads to an increased glucose intolerance and heightened insulin resistance (2, 3).

The effects of hypothyroidism on glucose metabolism appear to be complex and even puzzling since clinical findings point into seemingly contrasting directions: on the one hand, hypothyroidism is viewed as a potential cause of hypoglycaemia and, thus, should be controlled in cases of unexplained low blood glucose (4) and on the other hand, some but not all studies found an association of hypothyroidism with insulin resistance (5). Individuals with low thyroid function and prediabetes seem to be more likely to progress to type 2 diabetes compared to those with prediabetes and thyroid hormone levels in the reference range (6). The effects of subclinical hypo- or hyperthyroidism on glucose metabolism can be expected to be subtle but are less examined in previous studies, which also reported conflicting results (7, 8). Moreover, it is unclear if age-associated functional changes in the thyroid gland may modify the effect of hyper- or hypothyroidism on glucose metabolism. Since many study populations covered only a small age range, this has not hitherto been studied thoroughly.

The goal of this study was to investigate the association of thyrotropin (TSH), fT4, and fT3 with insulin resistance and disturbances in glucose metabolism in a large combined cohort of two cross-sectional epidemiological studies: the Study of Health in Pomerania (SHIP)-TREND-0 and the Berlin Aging Study II (BASE-II). A second aim was to examine potential effect modifications by age on these associations.

## Materials and methods

### Study population

SHIP-TREND-0 is a population-based cohort study conducted in the Northeast of Germany between 2008 and 2012 (9). For SHIP-TREND-0, a random, age- and sex-stratified sample of 8826 eligible subjects was drawn from population registries, of which 4420 subjects aged 20–80 years participated (net response 50.1%). All participants gave informed written consent. SHIP-TREND-0 followed the recommendations of the Declaration of Helsinki and was approved by the Ethics Committee of the University of Greifswald (Approval No. BB 39/08).

The BASE-II cohort was drawn as a convenience sample from the greater Berlin metropolitan area. For the medical part of the study, 2171 participants (~75% aged 60–84 years and ~25% aged 20–35 years) were recruited (10) and baseline assessment took place between 2009 and 2014. BASE-II is a longitudinal observational study to investigate factors associated with ‘healthy’ or ‘unhealthy’ aging. Study details have been described previously in detail (11, 12). All participants gave written informed consent and the Berlin Aging Study II was approved by the Ethics Committee of the Charité – Universitätsmedizin Berlin (Approval No. EA2/029/09) and registered with the German Clinical Trials Register (DRKS00009277).

From the present analysis, we excluded 614 individuals because of known diabetes and concomitant antidiabetic medication, 939 individuals due to missing data in laboratory values (glucose or insulin at baseline or after 2 h in the oral glucose tolerance test), 856 individuals because of a fasting period below 8 h before blood sampling, and 36 individuals due to systemic glucocorticoid therapy.

Altogether 4193 participants from the studies SHIP-TREND-0 (2381 participants) and BASE-II (1776 participants) were included in this cross-sectional analysis.

### Assessments

In SHIP, smoking status was assessed in a computer-assisted interview. In BASE-II, information on smoking status was taken from the medical history recorded by a study physician. Smoking status was categorized in the three categories: current smokers, former smokers, and never smokers. Information on pre-existing diabetes was taken from the medical history and assessed by interview.

In both the studies, body weight was measured in light clothes with a portable electronic scale to the nearest 0.1 kg and height was determined to the nearest 0.1 cm by using an electronic weighing and measuring station (seca 764, seca, Hamburg, Germany). Weight and height were used for calculating the BMI (weight (kg)/height (m)^2^).

Blood samples were taken as fasting blood samples and analysed in the local core laboratories using standardized protocols. Serum TSH, fT3, and fT4 levels were analysed by electrochemiluminescence immunoassays (BASE-II: cobas immunoassay systems, Roche Diagnostics; SHIP-TREND-0: Dimension Vista® System Flex® reagent cartridge, Siemens Healthcare Diagnostics Inc.). The analytical measuring range in BASE-II was 0.005–100 µIU/mL, 0.5–100 pmol/L, 0.6–50 pmol/L for TSH, fT4, and fT3, respectively. The analytical measuring range in SHIP-TREND-0 was 0.005–100 mIU/mL, 0.1–8.0 ng/dL, 0.50–30.00 pg/mL for TSH, fT4, and fT3, respectively. TSH levels were categorized in the three categories: TSH in the reference range, high TSH, and low TSH according to the established reference limits of 0.27–4.20 mU/L in BASE-II and 0.49–3.29 mIU/L in SHIP-TREND-0 (13).

In BASE-II, glucose concentration was determined by photometry, HbAlc by ion-exchange HPLC and serum insulin levels by chemiluminescence immunoassay (Roche elecsys, Roche Diagnostics). In SHIP-TREND-0, plasma glucose levels were measured using a hexokinase method (Dimension Vista 1500, Siemens Healthcare Diagnostics) and serum insulin values were assessed by an electrochemiluminescence immunoassay (ADVIA Centaur, Siemens Healthcare Diagnostics). HbA1c concentrations were determined by HPLC (Bio-Rad Diamat). A standardized oral glucose tolerance test (OGTT) was conducted and levels of insulin and glucose were measured at baseline and after 2 h in both studies. Impaired fasting glucose (IFG) and impaired glucose tolerance (IGT) as well as a formerly unknown diabetes were determined according to the criteria of the American Diabetes Association (ADA) (14).

In both studies, insulin resistance was calculated using the fasting glucose and insulin levels in the homeostasis model of insulin resistance (HOMA-IR) (15) as fasting glucose (mg/dL) × fasting insulin (mU/mL)/405. Insulin sensitivity was estimated by calculating the insulin sensitivity index (ISI) based on the work of Matsuda *et al.* (16) with measurements of glucose and insulin at baseline and 120 min after glucose challenge in the OGTT.

### Statistical analyses

Characteristics of the study population are provided as median, 25th, and 75th percentile for continuous variables or as absolute numbers and percentages for categorical variables stratified by study. In the pooled population of SHIP-TREND-0 and BASE-II, associations of thyroid biomarkers with continuous parameters of glucose metabolism were analysed by linear regression models adjusted for age, sex, smoking status, BMI, and study. Associations of thyroid biomarkers with prediabetes groups as proposed by the ADA (17) were analysed by multinomial logistic regression with adjustment for age, sex, smoking status, and study site. Interactions of serum TSH levels with age were tested in these regression models. A *P*  < 0.05 was considered statistically significant. All analyses were carried out using Stata16.0 (Stata Corporation, College Station, TX, USA).

## Results

The median age of the 4193 participants was 49 years in SHIP-TREND-0 and 67 years in BASE-II (Table 1). Altogether, 1928 participants were men and 2265 were women. SHIP-TREND-0 participants had a higher median BMI (27.0 kg/m^2^ vs 25.2 kg/m^2^ in BASE-II) and a higher proportion of unknown diabetes and prediabetes based on IFG and IGT than participants of BASE-II. A TSH below the reference range was found more often in SHIP-TREND-0 (7.5% vs 1.4% in BASE-II) while contrastingly a high TSH was observed less often in SHIP-TREND-0 (3.4% vs 6.7% in BASE-II). In both studies, participants reported taking thyroid medication (9.9% and 12.2% in SHIP-TREND-0 and BASE-II, respectively). Further characteristics of the study population are provided in Table 1.

**Table 1:** Characteristics of the study population stratified by study.

	SHIP-TREND-0 (*n* = 2381)	BASE-II (*n* = 1776)
Age, years	49 (39, 61)	67 (61, 70)
Males	1085 (45.6%)	843 (47.5%)
Smoking status		
Never	935 (39.3%)	861 (48.7%)
Former	862 (36.3%)	646 (36.5%)
Current	581 (24.4%)	262 (14.8%)
BMI, kg/m^2^	27.0 (24.2, 30.4)	25.2 (22.8, 28.0)
Waist circumference, cm	89 (79, 99)	92 (83, 100)
Systolic blood pressure, mmHg	125 (113, 136)	137 (125, 151)
Diastolic blood pressure, mmHg	76 (70, 83)	82 (75, 89)
HbA1c, %	5.2 (4.8, 5.5)	5.4 (5.1, 5.7)
Fasting glucose, mmol/L	5.4 (5.0, 5.8)	4.9 (4.6, 5.3)
2-h postload glucose, mmol/L	6.1 (5.1, 7.3)	5.4 (4.4, 6.5)
Fasting insulin, mU/L	9.4 (6.4, 13.9)	7.5 (5.2, 10.3)
2-h postload insulin, mU/L	52.4 (32.5, 94.4)	38.6 (23.7, 65.1)
Homeostasis model assessment (HOMA-IR)	2.24 (1.46, 3.47)	1.62 (1.11, 2.40)
Insulin sensitivity index (ISI)	5.8 (3.5, 9.3)	8.4 (5.4, 12.9)
Groups according to ADA criteria		
NGT	1332 (55.9%)	1418 (79.8%)
i-IFG	570 (23.9%)	158 (8.9%)
i-IGT	120 (5.0%)	85 (4.8%)
IFG + IGT	219 (9.2%)	72 (4.1%)
Unknown diabetes	140 (5.9%)	43 (2.4%)
TSH, mIU/L	1.18 (0.81, 1.66)	1.93 (1.30, 2.74)
fT3, pmol/L	4.73 (4.36, 5.11)	4.87 (4.45, 5.35)
fT4, pmol/L	13.3 (12.3, 14.5)	15.8 (14.3, 17.4)
Thyroid status		
Low TSH^a^	178 (7.5%)	24 (1.4%)
High TSH^a^	82 (3.4%)	118 (6.7%)
Intake of thyroid medication	236 (9.9%)	216 (12.2%)

Data are expressed as median, 25th, and 75th percentile (continuous data) or as absolute numbers and percentages (categorical data).

^a^According to the established reference limits of 0.27–4.20 mU/L in BASE-II and 0.49–3.29 mIU/L in SHIP-TREND-0.

i-IFG, impaired fasting glucose; i-IGT, impaired glucose tolerance; NGT, normal glucose tolerance.

### Associations of levothyroxine intake with thyroid biomarkers and glycaemic markers

Levothyroxine intake was significantly positively associated with fasting insulin (*P*  = 0.020) and HOMA-IR (*P*  = 0.025) but not with fasting glucose, 2-h postload glucose, and insulin or ISI. To account for potential effects of l-thyroxin on our results, we excluded all participants taking l-thyroxin from the multivariable analyses (*n*  = 424).

### Association of parameters of thyroid function with glycaemic markers

**Figure 1:**
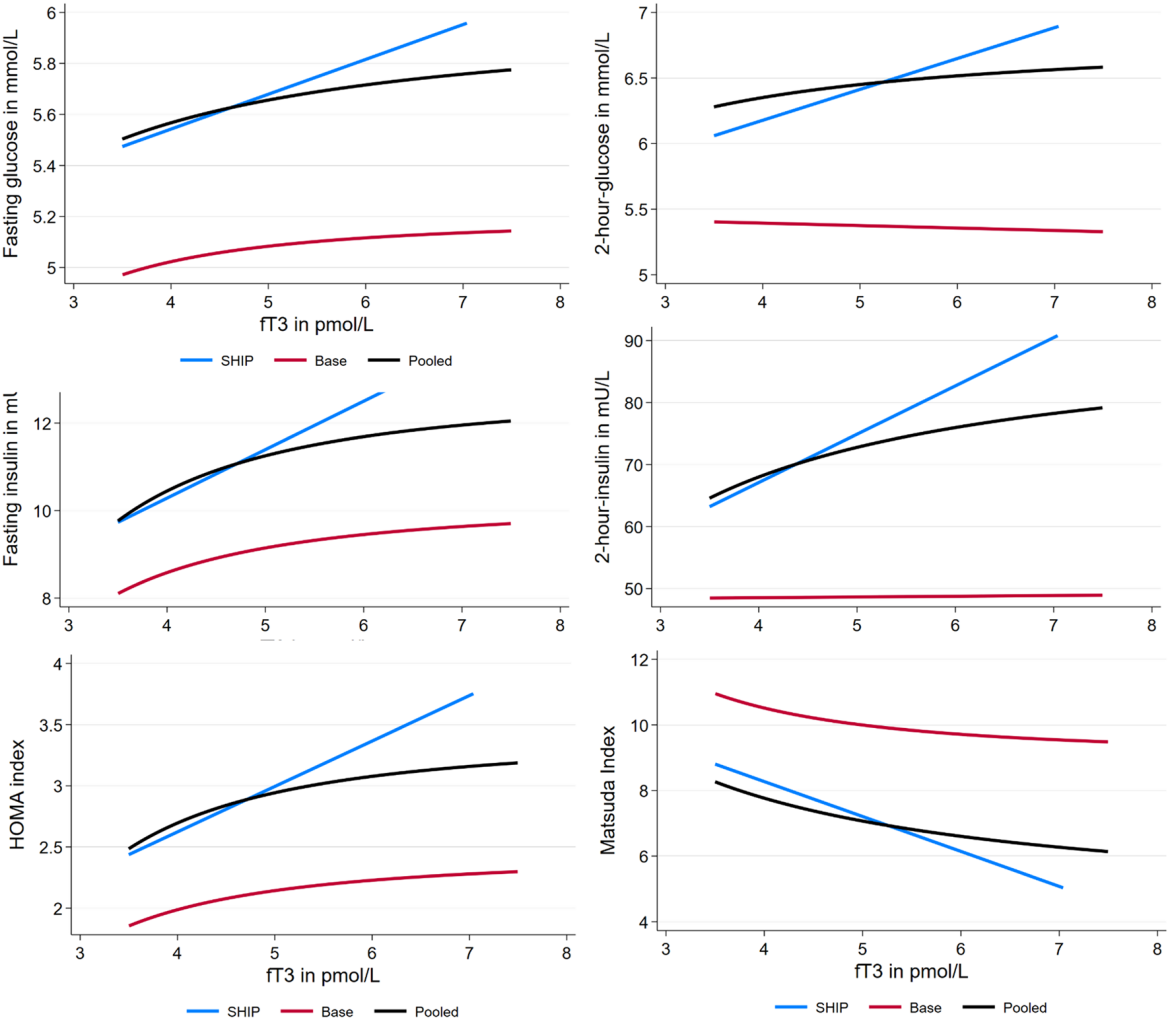
Associations of fT3 with glycaemic markers in SHIP-TREND-O, BASE-II, and the pooled population.

In the pooled sample of SHIP-TREND-0 and BASE-II, we analysed the associations of serum levels of TSH, fT3, and fT4 with fasting and 2-h postload glucose, fasting and 2-h postload insulin, HOMA-IR, and ISI in multivariable linear regression models. We found a positive association between TSH with 2-h postload insulin (β = 1.079, 95% CI = 0.026, 2.132) and positive associations of fT4 with fasting (β = 0.012, 95% CI= 0.002, 0.023) and 2-h postload glucose (β = 0.043, 95% CI = 0.013, 0.074). The results are summarized in Table 2. On the other hand, fT3 was significantly associated with all of the investigated markers except 2-h postload glucose. These relationships were predominantly non-linear and are displayed in Fig. 1. Serum fT3 levels were significantly positively associated with fasting glucose levels, fasting, and 2-h postload insulin levels as well as with the HOMA-IR. Accordingly, higher fT3 was associated with a lower ISI.

**Table 2:** Associations of serum TSH, fT3, and fT4 levels with glycaemic markers in the pooled population not taking l-thyroxin (*n*  = 3719).

	TSH, mIU/L, β (95% CI)	fT3, mIU/L, β (95% CI)	fT4, mIU/L, β (95% CI)
Linear regression: β (95% CI)			
Fasting glucose, mmol/L	−0.001 (−0.014, 0.013)	NL^a^	0.012 (0.002, 0.023)^a^
2-h postload glucose, mmol/L	0.013 (−0.025, 0.052)	NL (*P* = 0.075)	0.043 (0.013, 0.074)^a^
Fasting insulin, mU/L	−0.023 (−0.151, 0.106)	NL^a^	−0.026 (−0.125, 0.072)
2-h postload insulin, mU/L	1.079 (0.026, 2.132)^a^	NL^a^	0.185 (−0.628, 0.998)
Homeostatis model assessment (HOMA-IR)	−0.006 (−0.045, 0.033)	NL^a^	0.006 (−0.024, 0.036)
Insulin sensitivity index (ISI)	0.001 (−0.103, 0.105)	NL^a^	−0.020 (−0.100, 0.060)

Analyses are adjusted for age, sex, smoking status, BMI, and study.

^a^ *P*  < 0.05.

NL, non-linear relationship (see Fig. 1).

In the 427 individuals with l-thyroxin treatment, we observed no significant associations of TSH or fT4 with any of the glycaemic markers (Supplementary Table 1, see section on supplementary materials given at the end of this article). In this subgroup, serum fT3 levels were positively associated in a non-linear fashion with all glycaemic markers except fasting glucose. The non-linear associations were comparable to those in individuals not on l-thyroxin treatment.

### Association of parameters of thyroid function with prediabetes groups (according to ADA classification)

In multinomial logistic regression models, we investigated associations of TSH, fT3, and fT4 with IFG, IGT, the combination of IFG and IGT, and unknown diabetes. There were no significant associations of TSH with prediabetes groups. We observed a slightly higher relative risk for IFG with higher fT3 (RR 1.09, 95% CI = 1.02, 1.18). The association of fT3 with the IFG + IGT group barely missed statistical significance. Contrary to fT3, a higher fT4 was associated with a lower risk for IFG (RR 0.94, 95% CI = 0.89, 0.98). Furthermore, fT4 was positively associated with unknown diabetes. The results are summarized in Table 3.

**Table 3:** Associations of serum TSH, fT3, and fT4 levels with (pre)diabetes groups in the pooled population not taking l-thyroxine (*n*  = 3719).

(Pre)diabetes groups	TSH, mIU/L, β (95% CI)	fT3, mIU/L, β (95% CI)	fT4, mIU/L, β (95% CI)
Multinomial logistic regression: relative risk ratio (95% CI)			
NGT	Base outcome	Base outcome	Base outcome
i-IFG	0.96 (0.88, 1.04)	1.09 (1.02, 1.18)^a^	0.94 (0.89, 0.98)^a^
i-IGT	1.04 (0.99, 1.10)	1.00 (0.89, 1.14)	1.02 (0.95, 1.10)
IFG + IGT	0.96 (0.84, 1.09)	1.09 (0.99, 1.20)	1.05 (0.98, 1.13)
Unknown diabetes	0.97 (0.83, 1.12)	1.05 (0.90, 1.22)	1.09 (1.01, 1.19)^a^

Analyses are adjusted for age, sex, smoking status, BMI, and study.

^a^*P*  < .05.

i-IFG impaired fasting glucose; i-IGT impaired glucose tolerance; NGT, normal glucose tolerance.

### Interaction of age on the association of thyroid function with glycaemic markers

**Figure 2:**
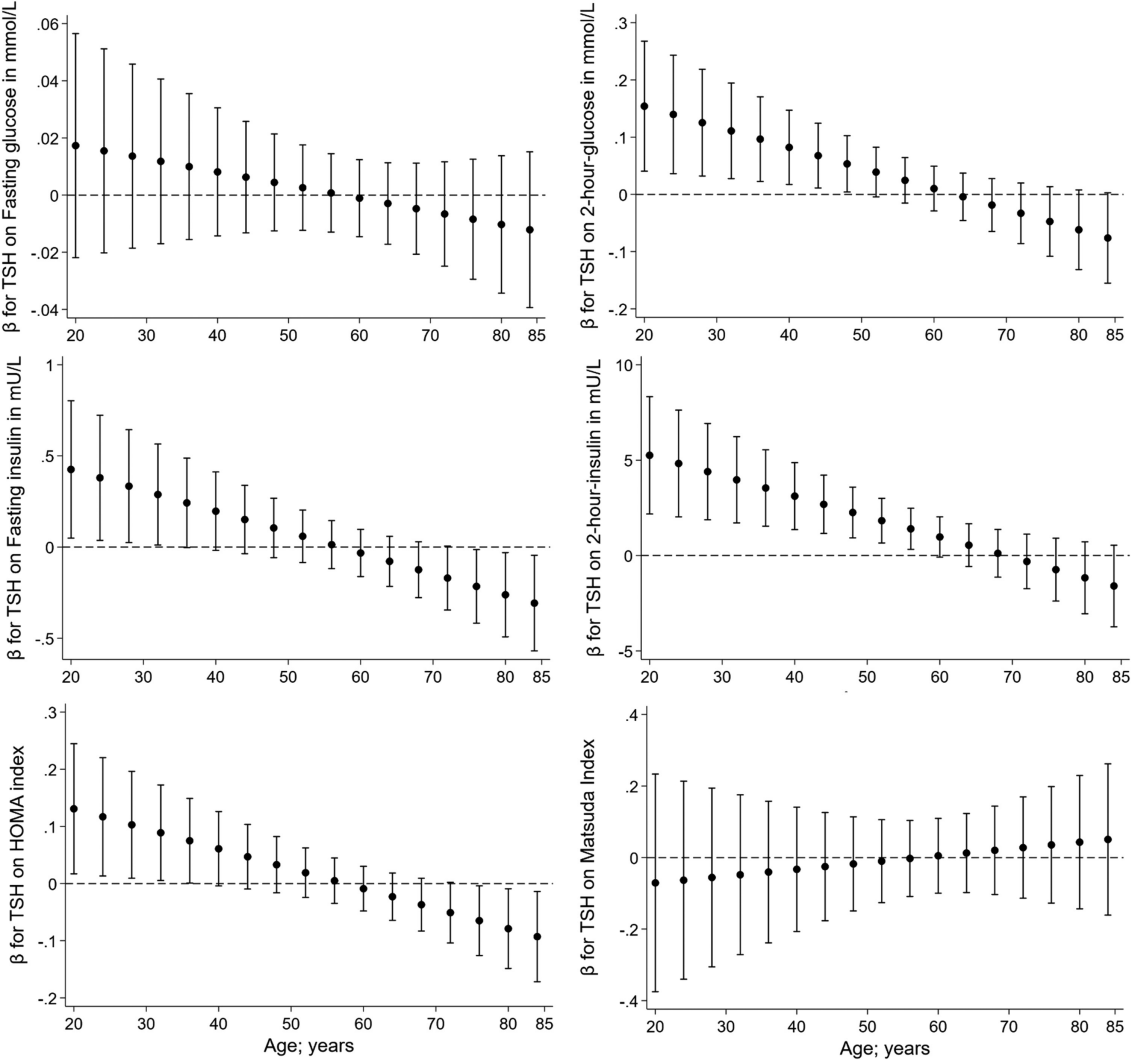
Interactions of TSH with age on glycaemic markers with depiction of each of the age-specific β coefficients with their 95% CI plotted against age.

**Figure 3:**
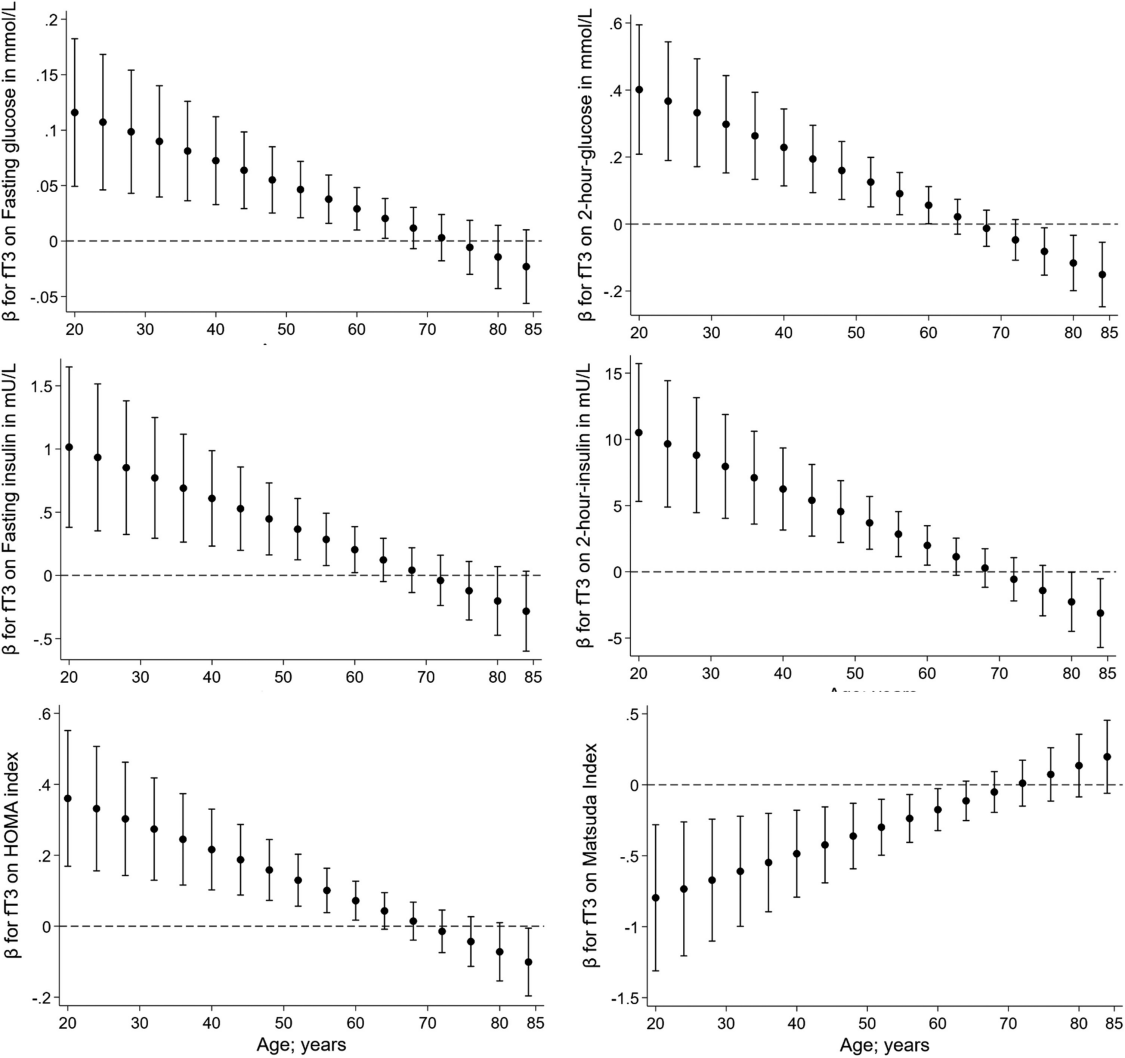
Interactions of fT3 with age on glycaemic markers with depiction of each of the age-specific β coefficients with their 95% CI plotted against age.

**Figure 4:**
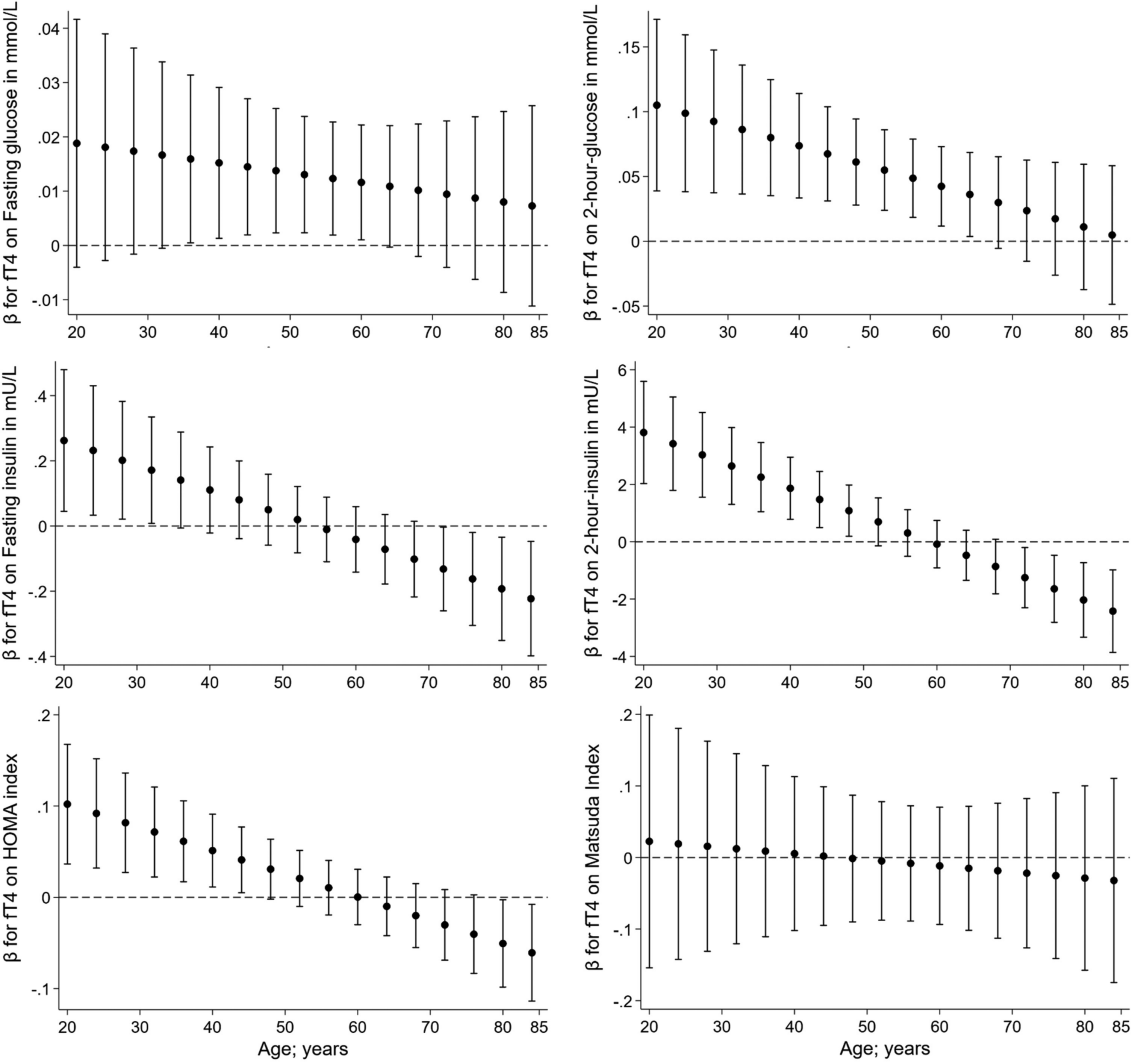
Interactions of fT4 with age on glycaemic markers with depiction of each of the age-specific β coefficients with their 95% CI plotted against age.

In the following analyses, we investigated whether age was an effect modifier for the association of thyroid markers with parameters of glucose and insulin metabolism. We observed many significant interactions of TSH, fT3, and fT4 with age on markers of prediabetes (Table 4). Based on the interaction models, we plotted the age-specific β coefficients with their 95% CI against age in Figs 2, 3 and 4. While there were significant interactions of fT3 with age on all markers of prediabetes, fT3 was also the only marker showing a significant interaction with age on the ISI. Moreover, TSH and age were interacting significantly on all other markers except the fasting glucose and ISI, whereas fT4 was interacting with age only on fasting and 2-h postload insulin and HOMA-IR. The interactions of TSH with age on the prediabetes markers were showing into the same – but functionally opposite – direction as fT3 and fT4. Most of the associations between thyroid markers and markers of prediabetes were significant in young and middle-aged individuals but not in the older participants.

**Table 4:** Interactions of thyroid biomarkers with age on glycaemic markers in the pooled population.

	TSH, mIU/L	fT3, mIU/L	fT4, mIU/L
Fasting glucose, mmol/L	0.341	0.003	0.525
2-h postload glucose, mmol/L	0.010	<0.001	0.057
Fasting insulin, mU/L	0.014	0.004	0.005
2-h postload insulin, mU/L	0.005	<0.001	<0.001
Homeostatis model assessment (HOMA-IR)	0.013	<0.001	0.002
Insulin sensitivity index (ISI)	0.612	0.006	0.697

Data are expressed as *P*-value for the interaction terms derived from linear regression.

### Association of parameters of serum fT3 levels with glycaemic markers stratified by age groups

For better understanding of our data and the age-specific effects on the association of thyroid function with the glycaemic markers, we added a sensitivity analysis with a stratification by age-specific subgroups (Supplementary Table 2). Here, we replicated the main findings of the associations of serum fT3 levels with glycaemic markers in groups of participants aged below 40 years and below 50 years. In the age group of 50 years and above, fT3 was not significantly associated with any of the glycaemic markers. Likewise, in the age group of 70 years and above, no significant associations of serum fT3 levels with the glycaemic makers were observed.

## Discussion

Our analyses of two cross-sectional epidemiologic German studies demonstrated consistent associations of fT3 levels with glycaemic markers and increased insulin resistance. Moreover, we observed a strong effect of age on the interaction of thyroid function with glycaemic markers. Interestingly, most of the associations between thyroid markers and glycaemic markers were significant in young and middle-aged individuals up to an age of roughly 60 years but not in older participants.

Findings from previous studies examining the relation between thyroid function and glucose metabolism are conflicting. El Demellawy *et al.* examined 40 patients with newly diagnosed hypothyroidism in comparison to 40 healthy controls (18). Interestingly, they found elevated markers of insulin resistance including HOMA-IR in both the hypo- and hyperthyroid group. The TSH correlated positively and fT3 and fT4 inversely with HOMA-IR suggesting that hypothyroidism might be strongly associated with insulin resistance than hyperthyroidism. Accordingly, the same results have been reported by Kapadia *et al.* in a small cross-sectional study including eight euthyroid, eight hyperthyroid, and eight hypothyroid patients (19). In another comparable setting, Abdel-Gayoum *et al.* described increased parameters of insulin resistance in newly diagnosed subclinical and overt hypothyroidism (20). An association of subclinical hypothyroidism with insulin resistance was also reported in a study with 30 participants compared to age-matched euthyroid controls (5). Contrary to our study, fT3 was inversely associated with HOMA-IR. However, sample sizes of all these studies have been comparably small and not all analyses were adjusted for potential confounders such as age (20).

In a much larger study with 3148 patients, there was no difference in markers of insulin resistance between euthyroid and subclinically hypothyroid subjects (21). However, in that study, fT4 was inversely associated with fasting insulin and HOMA-IR after adjustment for age, sex, and BMI, whereas in our study, we did not find such an association. In 8452 participants of the Rotterdam study, a large longitudinal population-based cohort study, the risk for diabetes or for progression from prediabetes to diabetes increased with higher TSH levels and lower fT4 levels. In this study, age had no effect modification on these associations (6).

Whereas some studies on the topic have been small and many used surrogate parameters of insulin resistance (which has been the case in SHIP and BASE-II either), the sample size in the RISC (relationship between insulin sensitivity and cardiovascular disease) study was reasonably high with 940 participants (22). Moreover, a gold standard for determining insulin resistance was used with the euglycaemic–hyperinsulinaemic clamp method (22). In this cohort, higher fT3 levels, within the reference range, were consistently associated with insulin resistance (22). This is in line with our results. In longitudinal analyses in the RISC study, fT3 was also predictive for an increase in fasting glucose and decrease in insulin sensitivity and ß-cell glucose sensitivity at follow-up. The lack of any association of TSH with metabolic parameters of insulin resistance can be estimated as another similarity to our study, where TSH was only associated with 2-h postload insulin with regard to continuous variables.

An association of low fT3 and low total T3 levels with decreased HOMA-IR was found in a study based on National Health and Nutrition Examination Survey data, which can be interpreted in accordance with our results (23). Rezzonico *et al.* also found a moderate linear relationship between T3 and HOMA-IR in study participants who were either euthyroid or subclinically hyperthyroid due to levothyroxine intake or for endogenous causes (24).

In summary of the available study data, insulin resistance and dysglycaemia can occur in both hypo- and hyperthyroidism. Moreover, these effects seem not to be restricted to overt hypo- or hyperthyroidism but might encompass subclinical disorders or even alterations of hormone levels in the reference range. The pronounced associations of fT3 with markers of insulin resistance that was found in our study, however, seem biologically plausible. Even though recruitment of glucose transporters (GLUT), such as GLUT3 and GLUT4, is increased in hyperthyroidism potentially leading to more glucose disposal in peripheral tissues, this might be overruled by insulin antagonistic actions of thyroid hormones, such as an increased glucose output from the liver and intestinal glucose absorption, decreased muscle glycogen storage, or upregulated glycogenolysis (2, 3, 25, 26, 27). Moreover, an increased lipolysis leading to heightened insulin resistance or an increased insulin clearance may link higher levels of fT3 to impaired glucose metabolism (3, 25, 26, 28).

Concerning the influence of age on the associations of thyroid function with glycaemic markers, much is still left to hypothesizing. However, it is already well known that the endocrine system, including the thyrotropic axis, undergoes functional changes during ageing. Many of the changes concerning thyroid function and aging have been summarized by Gesing *et al.* in a concise review (29). Of note, subclinical hypothyroidism seems to be not necessarily associated with outcomes such as cognitive impairment, physical impairment, depression, or metabolic disturbances in older people as it is known with overt hypothyroidism or as can be found in people of younger age in subclinical hypothyroidism (29). On the other hand, subclinical hyperthyroidism seems to be more consistently associated with outcomes such as decreased bone mineral density, fractures, or mortality (29), although this is not confirmed by all studies (30). Moreover, fT3 and fT4 levels were found to be inversely associated with age and might be linked to longevity (31), but conversely, making interpretation more complex, at least low T3 levels can also be linked to severe extrathyroidal illness as in the low T3 syndrome. A healthy survivor bias may also have influenced our analyses. Some of the participants with a lower fT3 due to extrathyroidal illness could already have died leaving healthier participants as survivors left for the analyses.

In our study, most of the potentially inverse effects of higher fT3 levels on glucose homeostasis and insulin sensitivity are observed in young and middle-aged individuals but not in the older individuals. This was confirmed in the additional analyses with stratification by age groups. Thus, a slightly higher fT3 might even be beneficial in terms of glucose metabolism in higher age although this has to be confirmed in longitudinal studies. On the other hand, insulin resistance is determined by numerous other factors such as adipose tissue or cortisol and growth hormone levels that might be more crucial than thyroid function. Multiple comorbidities and polypharmacy might also play an additional and more decisive role in higher age and might overrule the effect of the predominantly subclinical thyroid dysfunction on glucose metabolism found in our study. Moreover, fT3 has a short half-life of less than 24 h and might reflect predominantly short-term effects. Short- vs long-term effects might also explain the non-reciprocal effects which have been seen between TSH on the one hand and fT3 and fT4 on the other hand. Of note, recent data suggest that thyroid hormone levels may be strongly associated with clinical parameters as are TSH levels (32). The association of T3 and fT3 levels with metabolic parameters seem to be strong compared to TSH and thus in this context thyroid function might be better assessed with measuring free thyroid hormones (32).

One of the limitations of our study is the use of different laboratory assays for the measurement of TSH, fT3, and fT4. Although both were electrochemiluminescence immunoassays used in quality-driven laboratories, we cannot preclude a potential effect that biases the results. This must also be considered with regard to other parameters such as insulin. The strength of this study lies in its large sample size and the combination of two different cohorts covering a large age range that is important since thyroid disease can occur all over the lifespan. Also, the measurement of fT3 seems to be not part of many other comparable studies, which measured only TSH and fT4.

In summary, we found a consistent association between fT3 and markers of glucose metabolism. An increased fT3 might be a risk factor for insulin resistance but this effect might be restricted to younger and middle-aged individuals. This is noteworthy because from a clinical point of view, early detection of impaired glucose metabolism can prove to be decisive in terms of disease prevention. Moreover, our results may serve as another example that the investigation of associations of thyroid function with clinical parameters often demands the consideration of age-specific aspects. However, our data are only cross-sectional and cannot determine causality. Further studies are therefore warranted on this subject.

## Supplementary Material

Supplementary Table 1. Associations of serum TSH, fT3 and fT4 levels with glycemic markers in the pooled population taking L-Thyroxine (n=427)

## Declaration of interest

The authors declare that there is no conflict of interest that could be perceived as prejudicing the impartiality of this study.

## Funding

The BASE-II research project (Co-PIs are Lars Bertram, Ilja Demuth, Denis Gerstorf, Ulman Lindenberger, Graham Pawelec, Elisabeth Steinhagen-Thiessen, and Gert G. Wagner) is supported by the German Federal Ministry of Educationhttp://dx.doi.org/10.13039/100010002 and Research (Bundesministerium für Bildung und Forschung, BMBF) under grant numbers #16SV5536K, #16SV5537, #16SV5538, #16SV5837, #01UW0808, 01GL1716A, and 01GL1716B. Another source of funding is the Max Planck Institute for Human Development, Berlin, Germany. Additional contributions (e.g. equipment, logistics, personnel) are made from each of the other participating sites. The Study of Health in Pomerania is part of the Community Medicine Research Network of the University Medicine Greifswald, which was funded by the German Federal Ministry for Education and Research, the Ministry for Education, Research and Cultural Affairs, and the Ministry for Social Affairs of the State Mecklenburg-West Pomerania.

## Author contribution statement

H V, M N, and E S T were in charge of overall direction and planning. D S, N B, S S, M M, and T I performed the research, collected and analyzed data. M D, I D, and J S contributed to the interpretation of the results. D S and T I wrote the manuscript. All authors discussed the results and contributed to the final manuscript.
