# Supplementary material for: Association of thyroid function with insulin resistance: data from two population-based studies
Source: Eur Thyroid J. 2022 Jan 27;11(2):e210063. doi: 10.1530/ETJ-21-0063 (PMC8963174; doi:10.1530/ETJ-21-0063)
Supplement: Supplementary Table 1. Associations of serum TSH, fT3 and fT4 levels with glycemic markers in the pooled population taking L-Thyroxine (n=427) [file supplementary_table_1.pdf]

Supplementary Table 1. Associations of serum TSH, fT3 and fT4 levels with glycemic markers in the pooled population taking L-Thyroxine (n=427)

	**TSH; mIU/L**	**fT3; mIU/L**	**fT4; mIU/L**
	**β (95%- CI)**	**β (95%- CI)**	**β (95%- CI)**
	**Linear regression: β (95%- CI)**		
**Fasting glucose; mmol/L**	0.014 (-0.009; 0.036)	0.003 (-0.022; 0.028)	-0.009 (-0.025; 0.006)
**2-hour postload glucose; mmol/L**	0.029 (-0.049; 0.106)	NL*	0.004 (-0.049; 0.058)
**Fasting insulin; mU/L**	-0.066 (-0.311; 0.178)	NL*	-0.155 (-0.324; 0.015)
**2-hour postload insulin; mU/L**	1.089 (-3.197; 1.018)	NL*	-0.636 (-2.139; 0.867)
**Homeostatis model assessment (HOMA-IR)**	-0.007 (-0.082; 0.068)	NL*	0.044 (-0.095; 0.007)
**Insulin sensitivity index (ISI)**	0.015 (-0.157; 0.188)	NL*	0.043 (-0.077; 0.163)

CI confidence interval; NL non-linear relationship;
analyses are adjusted for age, sex, smoking status, body mass index and study
*p<0.05

Supplementary Table 2. Associations of serum fT3 levels with glycemic markers in age-specific subgroups of the pooled population

	**Age < 40 years (n=994) β (95%- CI)**	**Age < 50 years (n=1480) β (95%- CI)**	**Age ≥ 50 years (n=2152) β (95%- CI)**	**Age ≥ 70 years (n=611) β (95%- CI)**
	**Linear regression: β (95%- CI)**			
**Fasting glucose; mmol/L**	0.05 (0.01; 0.09)*	0.10 (0.05; 0.14)*	0.01 (-0.01; 0.03)	0.01 (-0.02; 0.04)
**2-hour postload glucose; mmol/L**	0.15 (0.03; 0.27)*	0.29 (0.17; 0.41)*	-0.01 (-0.07; 0.05)	-0.01 (-0.12; 0.08)
**Fasting insulin; mU/L**	0.60 (0.18; 1.03)*	0.90 (0.50; 1.30)*	0.05 (-0.15; 0.26)	0.05 (-0.25; 0.34)
**2-hour postload insulin; mU/L**	4.25 (0.84; 7.66)*	7.01 (3.51; 10.50)*	0.40 (-1.25; 2.04)	0.57 (-1.88; 3.01)
**Homeostatis model assessment (HOMA-IR)**	0.17 (0.07; 0.28)*	0.31 (0.19; 0.43)*	0.02 (-0.05; 0.07)	0.00 (-0.09; 0.10)
**Insulin sensitivity index (ISI)**	-0.93 (-1.65; -0.29)*	-1.19 (-1.70; -0.68)*	-0.03 (-0.16; 0.10)	-0.08 (-0.28; 0.12)

CI confidence interval;
analyses are adjusted for age, sex, smoking status, and study
*p<0.05
